# Serum Soluble Toll-Like Receptor 4 is a Predictive Biomarker for Acute Exacerbation and Prognosis of Idiopathic Pulmonary Fibrosis: A Retrospective Study

**DOI:** 10.1007/s00408-025-00800-y

**Published:** 2025-03-12

**Authors:** Erika Kitadai, Kakuhiro Yamaguchi, Hiroshi Iwamoto, Kiyofumi Shimoji, Shinjiro Sakamoto, Yasushi Horimasu, Takeshi Masuda, Taku Nakashima, Shinichiro Ohshimo, Hironobu Hamada, Noboru Hattori

**Affiliations:** 1 Department of Molecular and Internal Medicine, Graduate School of Biomedical and Health Sciences, Hiroshima University, 1-2-3 Kasumi, Minami-ku, Hiroshima, 734-8551 Japan; 2 Department of Emergency and Critical Care Medicine, Graduate School of Biomedical and Health Sciences, Hiroshima University, Hiroshima, Japan; 3 Department of Physical Analysis and Therapeutic Sciences, Graduate School of Biomedical and Health Sciences, Hiroshima University, Hiroshima, Japan

**Keywords:** Acute exacerbation, Prognosis, Idiopathic pulmonary fibrosis, Soluble toll-like receptor 4, Monocyte

## Abstract

**Purpose:**

Toll-like receptor 4 (TLR4) is a transmembrane receptor promoting pro-inflammatory signalling, that is associated with the pathogenesis of pulmonary fibrosis. TLR4 is abundantly expressed on monocytes and the acceleration of TLR4 signalling induces the secretion of soluble TLR4 isoforms (sTLR4) in circulation. The aim of study was to evaluate the association of serum levels of sTLR4 with acute exacerbation (AE) and prognosis of patients with idiopathic pulmonary fibrosis (IPF).

**Methods:**

This retrospective cohort study included 97 patients with IPF and 76 healthy participants. The association of serum sTLR4 levels with the onset of AE and the prognosis in 97 patients with IPF was analyzed.

**Results:**

No significant difference in sTLR4 serum level was observed between the patients with IPF and healthy participants. Kaplan–Meier curves showed that patients with sTLR4 ≥ 2.2 ng/mL had a significantly higher incidence of AE-IPF and a significantly lower 5-year survival rate. Univariate and multivariate Cox hazard analyses demonstrated that sTLR4 ≥ 2.2 ng/mL was significantly associated with higher incidence of AE and poorer survival. In an exploratory analysis, a weak correlation was observed between sTLR4 levels and monocyte counts, and the incidence of AE-IPF was the highest in the patients with sTLR4 ≥ 2.2 ng/mL and monocyte counts ≥ 381/μL.

**Conclusion:**

High sTLR4 level is associated with an increased incidence of AE-IPF and poor prognosis in patients with IPF. The combination of sTLR4 level and monocyte count might be used to stratify patients with IPF according to the risk for AE via reflecting monocyte activation.

**Supplementary Information:**

The online version contains supplementary material available at 10.1007/s00408-025-00800-y.

## Introduction

Idiopathic pulmonary fibrosis (IPF) is a progressive and irreversible fibrotic lung disease of unknown etiology. “Acute exacerbation” is an acute complication of IPF (AE-IPF) that leads to death in approximately 40% cases [1, 2]. Although the pathogenesis of AE-IPF is still unclear, the enhanced expression of damage-associated molecular patterns, such as high-mobility group box 1 (HMGB1) and S100 proteins, is associated with increased incidence and poor prognosis of acute exacerbation (AE) in patients with IPF. Higher level of serum HMGB1 at diagnosis is associated with earlier onset of AE-IPF [3]. Additionally, circulatory levels of HMGB1, S100A8, and S100A9 at the onset of AE-IPF are significantly increased compared to the levels at diagnosis of IPF, and the high levels also result in poorer survival in patients with AE-IPF [3–5]. Thus, inflammatory signalling accelerated by HMGB1 and S100 proteins is believed to promote the development of AE and aggravate the severity of AE-IPF.

Toll-like receptor 4 (TLR4) is a transmembrane receptor abundantly expressed in monocytes and is one of the receptors for HMGB1 and S100 proteins [3, 6–10]. The interaction between TLR4 and TLR4 ligands such as HMGB1 increases inflammatory signalling, thereby contributing to organ fibrosis including pulmonary fibrosis [11–16]. On the other hand, TLR4 signalling also plays an important role in maintaining lung homeostasis [17]. Therefore, excessive interaction between TLR4 and TLR4 ligands is believed to promote the progression of fibrotic lung diseases [17].

Soluble TLR4 (sTLR4) is a soluble isoform of TLR4 without an intracellular signalling domain, that circulates in the blood [18]. sTLR4 is known to be secreted by acceleration of TLR4 signalling. In a previous in vitro study, lipopolysaccharide promotes TLR4 signalling, thereby secreting sTLR4 from mouse macrophage cell line RAW264.7 [19]. Additionally, sTLR4 levels are elevated in patients with bacterial infections [20]. It has also been reported that patients with non-small cell lung cancer (NSCLC) have higher levels of sTLR4 compared to healthy participants [21]. Moreover, higher levels of sTLR4 before treatment are associated with a higher incidence of radiation pneumonitis in patients with NSCLC [21]. These data indicate that sTLR4 can be used as a blood biomarker associated with aggravated inflammatory condition and lung damage. However, whether circulating levels of sTLR4 are associated with AE development and/or IPF progression remains unclear.

Therefore, we hypothesized that high level of sTLR4 is associated with the progression of IPF and/or the development of AE-IPF by reflecting excessive acceleration of TLR4 signalling. This study aimed to explore whether serum levels of sTLR4 could predict AE-IPF and prognosis of IPF.

## Methods

### Participants and Study Design

This retrospective cohort study screened 109 patients with IPF, who were treated at Hiroshima University Hospital between 2005 and 2020. Seven patients whose samples were collected during or after AE-IPF occurrence or infection were excluded. This study also excluded four patients who did not undergo pulmonary function tests and one patient whose serum sTLR4 level was out of the measurable range and could not be measured reproducibly using diluted samples. Finally, this study enrolled 97 patients with IPF, and the association of serum sTLR4 levels with AE and prognosis of IPF was analyzed. IPF was diagnosed according to the criteria of the American Thoracic Society/European Respiratory Society [22]. AE-IPF was diagnosed according to an International Working Group Report [1]. As healthy controls, 76 participants who underwent health examination and had no respiratory disease diagnosed through chest radiograph, pulmonary function tests, and self-reporting were enrolled. This study was approved by the Ethics Committee of Hiroshima University Hospital (M326) and conducted in accordance with the Declaration of Helsinki. All patients provided written informed consent and permitted the use of their samples.

### Data Collection and Measurement of Serum sTLR4

Patients’ clinical records were retrospectively reviewed. We obtained information regarding patient characteristics such as age, sex, smoking history, and pulmonary function tests.

Serum samples were collected at the first visit or before treatment. Serum samples were stored at − 80 ℃. Serum sTLR4 levels were measured using a TLR4 Enzyme-Linked Immunosorbent Assay kit (Invitrogen, Waltham, Massachusetts, The United States).

### Statistical Analysis

Data are presented as median (interquartile range). When differences among groups were examined, the Mann–Whitney U test was used to compare continuous variables and the chi-square test to compare nominal variables. The onset of AE-IPF and prognosis were evaluated using the Kaplan–Meier approach and log-rank test. Patients were censored at the time of loss to follow-up or completion of 5 years of follow-up. Death before AE-IPF was defined as censored when the association between sTLR4 and the onset of AE-IPF was analyzed. Cox proportional hazards analysis was performed to identify significant predictors of AE-IPF and prognosis over five years. Receiver operating characteristic (ROC) curve analysis was performed to determine the optimal cut-off values of serum sTLR4 and monocyte count for predicting AE-IPF occurrence within 5 years. Correlations between variables were ascertained using Spearman’s correlation coefficients. All statistical analyses were performed using the JMP Pro 18.1.0 software (SAS Institute Japan Co., Ltd., Tokyo, Japan). Statistical significance was set at *P* < 0.05.

## Results

### Clinical Characteristics

We analyzed 97 patients with IPF and 76 healthy participants. The median follow-up time in the patients with IPF was 40.1 months (21.4 − 62.3 months). The main characteristics of the patients and healthy participants are shown in Table 1 and Supplementary Information S1. The patients with IPF were significantly older than healthy participants (68.0 years and 61.5 years, *P* = 0.024). No significant differences in sex, body mass index (BMI), or pack-year smoking history were observed between patients with IPF and healthy participants. The patients with IPF had a significantly lower forced vital capacity (FVC) than healthy participants (74.3% vs. 95.0%, *P* < 0.0001). No significant differences in serum sTLR4 levels were observed between the patients with IPF and the healthy participants (1.0 ng/mL [0.7 − 1.8] and 1.2 ng/mL [0.8 − 3.1], *P* = 0.22; Supplementary Information S2).

**Table 1:** Clinical characteristics of the study participants

	All patients with IPF (*n* = 97)	sTLR4		Monocyte	
		< 2.2 ng/mL (*n* = 76)	≥ 2.2 ng/mL (*n* = 21)	< 381 /uL (*n* = 48)	≥ 381 /uL (*n* = 49)
Age, years	68.0 (62.0 − 74.0)	68.0 (61.2 − 74.0)	69.0 (64.5 − 76.0)	68.0 (62.3 − 75.5)	68.0 (61.5 − 73.5)
Sex, male/female	86/11	66/10	20/1	39/9	47/2*
BMI, kg/m^2^	23.7 (21.6 − 26.2)	24.1 (21.4 − 26.7)	23.5 (22.5 − 24.7)	23.5 (21.5 − 26.8)	24.0 (22.0 − 25.5)
Smoking history, pack-years	32.8 (15.9 − 49.5)	32.0 (10.0 − 48.0)	40.0 (27.0 − 60.0)	33.3 (15.0 − 50.0)	32.5 (19.1 − 47.3)
FVC, %predicted	74.3 (64.1 − 88.1)	73.4 (61.8 − 86.5)	76.4 (67.1 − 89.8)	75.0 (64.9 − 86.9)	74.2 (61.8 − 88.6)

Data are shown as median (Interquartile Range)

BMI, body mass index; FVC, forced vital capacity; IPF, idiopathic pulmonary fibrosis; sTLR4, soluble toll-like receptor 4;

*p < 0.05, Pearson chi-square test compared with patients with a low monocyte count

### Association of sTLR4 with AE-IPF and Prognosis of IPF

Twenty-three patients (23.7%) developed AE-IPF over 5 years. The 5-year survival rate was significantly lower in the patients with AE during the 5-year period compared to those without (*P* < 0.0001; Supplementary Information S3).

**Fig. 1:**
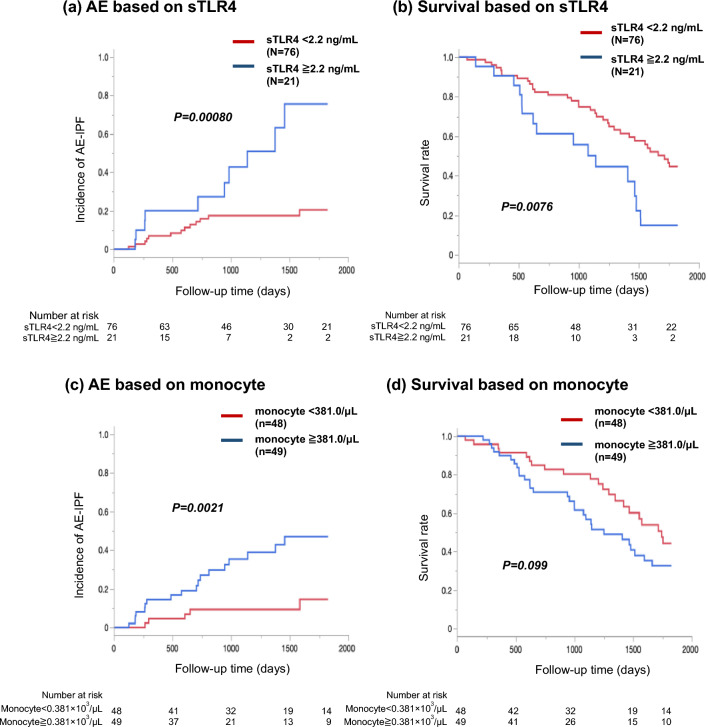
Incidence of AE-IPF and survival rate based on serum levels of sTLR4 and monocyte. In Figure a and b, red solid line indicates the patients with lower levels of sTLR4 (< 2.2 ng/mL), and blue solid line indicates the patients with higher levels (≥ 2.2 ng/mL). Among patients with IPF, those with higher levels of serum sTLR4 (≥ 2.2 ng/mL) showed a significantly higher incidence of AE-IPF (**a**) and poorer survival (**b**) than those with lower levels. In Fig. c and d, red solid line indicates the patients with lower levels of monocyte (< 381/μL), and blue solid line indicates the patients with higher counts (≥ 381/μL). Patients with higher monocyte counts (≥ 381/μL) showed a significantly higher incidence of AE-IPF (**c**) and tendency of poor survival (**d**) compared with patients with the lower counts in patients with IPF. IPF, idiopathic pulmonary fibrosis; sTLR4, soluble Toll-like receptor 4; AE, acute exacerbation

The optimal cut-off level of sTLR4 for predicting AE-IPF was 2.2 ng/mL (sensitivity, 43.5%; specificity, 85.1%), as identified by ROC curve analysis. Kaplan–Meier curve analysis and log-rank tests revealed that sTLR4 levels ≥ 2.2 ng/mL were associated with a higher incidence of AE-IPF (*P* = 0.00080; Fig. 1a). No significant differences in clinical characteristics were observed between patients grouped by sTLR4 levels (Table 1).

Additionally, the association between sTLR4 levels and the survival rate was analyzed. Soluble TLR4 levels ≥ 2.2 ng/mL were associated with a lower survival rate in patients with IPF (*P* = 0.0076; Fig. 1b).

### Association Between Serum sTLR4 Levels and Baseline Characteristics

**Fig. 2:**
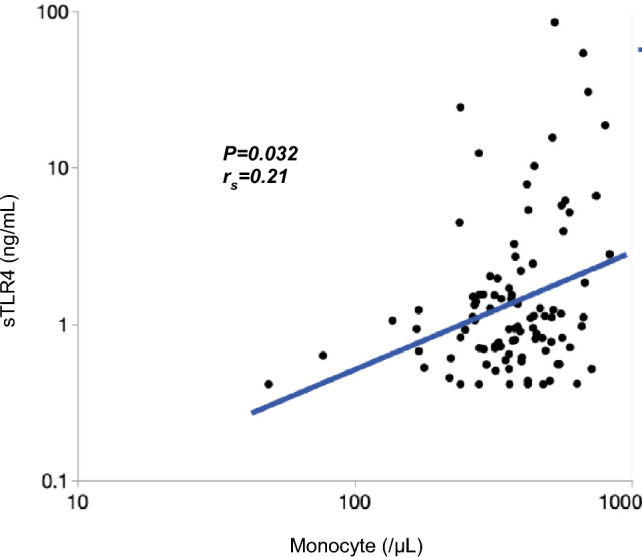
Association between sTLR4 levels and monocyte counts. Fig. 2 shows a weak correlation between serum levels of sTLR4 and monocyte counts (*P* = 0.032,* r*_*s*_ = 0.21). sTLR4, soluble Toll-like receptor 4

In patients with IPF, Spearman’s correlation coefficients revealed that serum levels of sTLR4 were positively, although weakly, correlated only with monocyte count as shown in Fig. 2 (*P* = 0.032, *r*_*s*_ = 0.21). However, there was no association between serum levels of sTLR4 and BMI, pack-years, FVC, white blood cell count, neutrophil count, lymphocyte count, platelet count, or C-reactive protein (CRP) levels (Supplementary Information S4).

### Association of Monocyte Count with AE-IPF and Prognosis of IPF

The optimal cut-off level of monocyte counts, as identified by ROC curve analysis, for predicting AE-IPF was 381/μL (sensitivity, 78.3%; specificity, 58.1%). Kaplan–Meier curve analysis and log-rank test revealed that monocyte levels ≥ 381/μL were associated with a higher incidence of AE-IPF (*P* = 0.0021; Fig. 1c). The survival rate tended to be lower in patients with high monocyte counts (*P* = 0.099; Fig. 1d). The clinical characteristics of patients grouped by monocyte levels are shown in Table 1. There were more men in the high monocyte level group, but no other significant differences were observed between the two groups.

### Cox Hazard Analyses to Identify the Independent Predictive Factors for AE-IPF and Poor Prognosis of IPF

In the univariate Cox proportional hazards models, serum sTLR4 ≥ 2.2 ng/mL was significantly associated with higher incidence of AE-IPF and shorter survival in the patients with IPF. On the other hand, monocyte count ≥ 381/μL was significantly associated with only higher incidence of AE-IPF (Table 2).

**Table 2:** Cox proportional hazards analysis to identify predictive factors for AE and prognosis of IPF

	AE			Survival		
	HR	95%CI	*P*-value	HR	95%CI	*P*-value
Univariate analysis						
Age, years	0.99	0.95 − 1.0	0.82	1.0	0.99 − 1.0	0.24
Sex, male	NA	NA	0.99	1.7	0.63 − 4.9	0.29
BMI, kg/m^2^	1.0	0.94 − 1.2	0.42	0.99	0.91 − 1.1	0.82
Smoking history, pack-years	1.0	0.99 − 1.0	0.35	1.0	0.98 − 1.0	0.43
FVC, % predicted	0.99	0.96 − 1.0	0.38	0.97	0.95 − 0.99	0.0010*
Monocyte, ≥ 381 /uL	4.2	1.6 − 11	0.046*	1.6	0.91 − 2.8	0.10
sTLR4, ≥ 2.2 ng/mL	3.8	1.7 − 8.8	0.0017*	2.3	1.2 − 4.2	0.0094*
Antifibrotic agent, +	0.93	0.40 − 2.1	0.86	1.5	0.83 − 2.7	0.18
Multivariate analysis						
FVC, % predicted	0.99	0.96 − 1.0	0.43	0.97	0.95 − 0.98	0.00060*
Monocyte, ≥ 381.0/uL	3.2	1.1 − 8.9	0.030*	1.4	0.74 − 2.5	0.33
sTLR4, ≥ 2.2 ng/mL	2.7	1.1 − 6.5	0.024*	2.2	1.2 − 4.3	0.017*

*AE* acute exacerbation, *BM*I body mass index, *CI* confidence interval, *FVC* forced vital capacity, *HR* hazard risk, *IPF* idiopathic pulmonary fibrosis, *NA* not available; *sTLR4* soluble toll-like receptor 4

*p < 0.05, Cox proportional hazards analysis

Multivariate Cox proportional hazards analysis included sTLR4 ≥ 2.2 ng/mL, monocyte count ≥ 381/μL, and FVC as explanatory variables because sTLR4 and monocyte count showed a significant association with AE and lower percentage of FVC was well known as a risk of AE and shorter survival. In this multivariate analyses, sTLR4 ≥ 2.2 ng/mL and monocyte count ≥ 381/μL were independently associated with higher incidence of AE-IPF. Additionally, sTLR4 levels and FVC, but not monocyte count, were independently associated with survival in patients with IPF.

### Risk Stratification of AE-IPF Based on the Combination of sTLR4 and Monocyte

In the exploratory analysis, the participants were divided into three groups according to the number of AE risks consisted of sTLR4 level and monocyte count identified in this study. Patients with two risks defined as group A (17 patients with sTLR4 level > 2.2 ng/mL and monocyte counts > 381.0/μL), patients with one risk defined as group B (36 patients with either higher levels of sTLR4 or higher monocyte counts), and patients with no risk defined as group C (44 patients with lower level of sTLR4 and lower monocyte counts). Group B included 4 patients with higher levels of sTLR4 and lower monocyte counts, and 32 patients with lower levels of sTLR4 and higher monocyte counts.

**Fig. 3:**
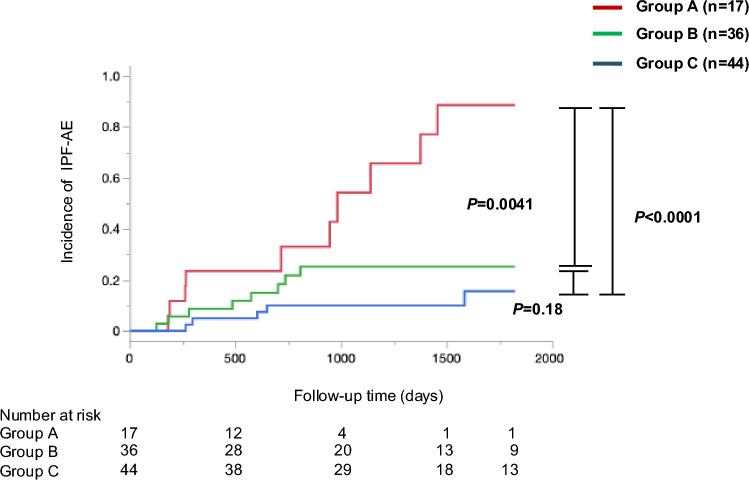
Incidence of AE-IPF based on the combination of sTLR4 and monocyte. In Fig. 3, three patients’ groups are defined based on sTLR4 level and monocyte count; Group A (red solid line), patients with sTLR4 level > 2.2 ng/mL and monocyte counts > 381/μL; Group B (green solid line), patients with either higher sTLR4 level or higher monocyte counts; Group C (blue solid line), patients with lower level of sTLR4 and monocyte counts. The incidence of AE-IPF is higher in Group A than that in Groups B and C. AE-IPF, acute exacerbation of idiopathic pulmonary fibrosis; sTLR4, soluble Toll-like receptor 4

The characteristics of the three groups are summarized in Supplementary Information S5. No significant differences in age, sex, BMI, smoking history, and FVC were observed among the three groups. Kaplan–Meier curve analysis and the log-rank test revealed that the incidence of AE-IPF in group A (higher levels of sTLR4 and monocyte) was higher than that in groups B and C (Fig. 3). Univariate and multivariate Cox proportional hazards analyses showed that patients in group A had the highest risk of developing AE-IPF (Table 3).

**Table 3:** Cox proportional hazards analysis for analysing the association between AE-IPF and the combination of sTLR4 and monocyte

	HR (vs Group A)	HR (vs Group B)	HR (vs Group C)
Univariate analysis			
Group A	ref	3.7 (1.4 − 9.5) *	7.2 (2.7 − 23.5) *
Group B	0.27 (0.11 − 0.70) *	ref	2.2 (0.70 − 6.6)
Group C	0.13 (0.043 − 0.37) *	0.46 (0.15 − 1.4)	ref
Multivariate analysis**			
Group A	ref	3.6 (1.4 − 9.3) *	7.8 (2.6 − 23.3) *
Group B	0.28 (0.11 − 0.71) *	ref	2.2 (0.70 − 6.6)
Group C	0.13 (0.043 − 0.38) *	0.46 (0.15 − 1.4)	ref

HR was described with 95% confidence interval

Group A, the patients with soluble toll-like receptor 4 (sTLR4) level higher than 2.2 ng/mL and monocyte level higher than 381.0/μL

Group B, the patients with either sTLR4 higher level or monocyte higher level

Group C, the patients with sTLR4 lower level and monocyte lower level

Data are shown as hazard risk (95% confidence interval)

AE, acute exacerbation; HR, hazard risk; IPF, idiopathic pulmonary fibrosis; ref, reference

*p < 0.05, Cox proportional hazards model

**Multivariate analysis was adjusted for FVC

## Discussion

This study demonstrated that high levels of serum sTLR4 are associated with an increased incidence of AE-IPF and poor prognosis in patients with IPF. To the best of our knowledge, this is the first study to report sTLR4 as a predictive blood marker for a higher incidence of AE-IPF and shorter survival of patients with IPF. This study also demonstrated that serum sTLR4 levels were positively, although weakly, correlated with monocyte counts, and that high monocyte counts were associated with an increased incidence of AE-IPF. In addition, the combination of sTLR4 and monocyte levels might help stratify patients with IPF who are at risk of AE.

High sTLR4 levels are associated with an increased incidence of AE-IPF and poor prognosis of IPF. sTLR4 is secreted in response to the activation of TLR4 signalling [19], and TLR4 is a transmembrane receptor abundantly expressed in monocytes [6–8]. TLR4 signalling pathway is involved in the activation of downstream cascades, such as nuclear factor-kappa B (NF-κB) and mitogen-activated protein kinase (MAPK), which are essential for the production of inflammatory cytokines and fibrotic factors [23]. This prolonged inflammatory response promotes tissue damage and fibrosis and may be involved in the progression of IPF. For example, TLR4 ligands include HMGB1 and S100 proteins [9, 10]. In patients with IPF, high serum HMGB1 levels at diagnosis are associated with an earlier onset of AE-IPF [3]. Additionally, circulatory levels of HMGB1, S100A8, and S100A9 at the onset of AE-IPF are significantly higher than those at the time of diagnosis. Moreover, high levels also result in poor survival in patients with AE-IPF [3–5]. Furthermore, AE-IPF is associated with shorter survival in patients with IPF, that was also confirmed in this study. These data support that activation of TLR4 signalling is associated with the development and aggravation of AE-IPF, thereby resulting in a poor prognosis of IPF. Therefore, reflected by TLR4 activation, serum sTLR4 level may be a potential predictive marker for AE-IPF and prognosis of IPF.

A high monocyte count is also associated with AE-IPF. Monocytes are known to contribute to the progression of pulmonary fibrosis. For example, although no significant difference in baseline monocyte counts was observed between patients with and without interstitial lung abnormalities (ILA), patients with ILA with higher monocyte counts show radiological progression of pulmonary fibrosis [24]. In patients with IPF, higher monocyte counts are associated with shorter transplant-free survival, increased risk of death from lung transplantation, decreased lung function, disease progression, and increased risk of hospitalization and death [25, 26]. Additionally, in line with our study, it has also been reported that higher monocytes counts are a risk factor for acute exacerbation of fibrotic interstitial pneumonia including IPF [27]. These data support the validity of our results showing the association between higher monocyte count and incidence of AE-IPF.

In addition, this study demonstrated a weak correlation between monocyte count and sTLR4 levels. Furthermore, this study showed that when sTLR4 levels and monocyte counts were combined, the risk of AE-IPF was highest in the group with both high sTLR4 level and monocyte count. Although the mechanism by which high monocyte counts promote pulmonary fibrosis remains unclear, monocytes appear to recruit damaged tissue to aid in repair. In patients with IPF, monocytes are reported to migrate to the lung and become alveolar macrophages, accelerating pulmonary fibrosis [28]. It should be noted that monocytes abundantly and physiologically express TLR4 [6–8], and activation of TLR4 signalling leads to secretion of sTLR4 and differentiation to macrophage [19, 29]. These findings suggest the concomitant presence of high sTLR4 levels and elevated monocyte counts may reflect an increased number of activated monocytes that promote differentiation into pulmonary macrophages, thereby accelerating pulmonary fibrosis. Therefore, the combination of high sTLR4 levels and elevated monocyte counts may be useful for identifying patients at a high risk of AE-IPF.

This study has several limitations. First, owing to the retrospective study design, baseline characteristics, especially age, differed between patients with IPF and healthy participants. Second, this study was unable to demonstrate a significant association between monocyte count and prognosis, as previously reported [26]. This could be attributed to the small sample size. Third, serial changes in sTLR4 serum levels could not be evaluated because of lacking serial samples, including those at the diagnosis of AE-IPF. Lastly, because this study was single cohort study evaluated in Japanese population, the association between sTLR4 and AE-IPF needs to be validated in other cohorts of different races. Further prospective studies with larger sample size are needed to confirm the clinical utility of sTLR4 in evaluating the risk of AE and prognosis in patients with IPF.

## Conclusions

sTLR4 may be a predictive marker for AE-IPF and the prognosis of IPF. Additionally, the combination of serum sTLR4 levels and monocyte counts may support the risk stratification of AE-IPF, although further confirmatory studies are necessary.

## Supplementary Information

Below is the link to the electronic supplementary material.

Supplementary file1 (PDF 144 KB)

## Data Availability

Data are available to interested researchers upon reasonable request to the corresponding author based on ethical approval.

## Abbreviations

AE: Acute exacerbation
BMI: Body mass index
FVC: Forced vital capacity
HMGB1: High mobility group box 1
IPF: Idiopathic pulmonary fibrosis
ROC: Receiver operating characteristic
sTLR4: Soluble toll-like receptor 4
TLR4: Toll-like receptor 4
WBC: White blood cell
