# Supplementary material for: Serum Soluble Toll-Like Receptor 4 is a Predictive Biomarker for Acute Exacerbation and Prognosis of Idiopathic Pulmonary Fibrosis: A Retrospective Study
Source: Lung. 2025 Mar 12;203(1):43. doi: 10.1007/s00408-025-00800-y (PMC11903555; doi:10.1007/s00408-025-00800-y)
Supplement: Supplementary file 1 — Supplementary file1 (PDF 144 KB) [file 408_2025_800_MOESM1_ESM.pdf]

Supplementary Information (SI)

S1. Serum sTLR4 levels in patients with IPF and healthy participants

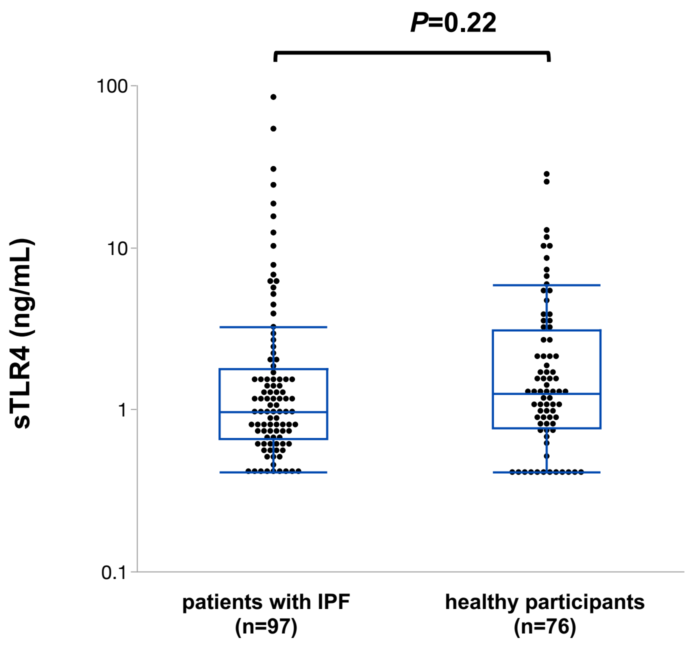

There were no significant differences in serum sTLR4 levels between patients with IPF and healthy participants (1.0 ng/mL [0.7−1.8] and 1.2 ng/mL [0.8−3.1], *P*=0.22, respectively). sTLR4, soluble toll-like receptor 4; IPF, idiopathic pulmonary fibrosis

S2: Clinical characteristics of the study participants

	All participants (n=173)	Patients with IPF (n=97)	Healthy participants (n=76)	*P*-value*
Age, years	66.0 (59.0−73.0)	68.0 (62.0−74.0)	61.5 (58.0−72.0)	0.024
Sex, male/female	149/24	86/11	63/13	0.28
BMI, kg/m^2	23.7 (21.4−25.7)	23.7 (21.6−26.2)	23.7 (21.2−25.4)	0.66
Smoking history, pack years	31.8 (8.1−50.0)	32.8 (15.9−49.5)	30.0 (0−57.5)	0.40
FVC, %predicted	82.8 (67.4−95.0)	74.3 (64.1−88.1)	95.0 (86.0−111.3) **	<.00010
sTLR4, ng/mL	1.1 (0.7−2.2)	1.0 (0.7−1.8)	1.2 (0.8−3.1)	0.22

Data are presented as the median (interquartile range)

* Comparison of patients with IPF and healthy participants

** There were 30 missing data in healthy participants.

BMI, body mass index; FVC, forced vital capacity; IPF, idiopathic pulmonary fibrosis; sTLR4, soluble toll-loke receptor 4

S3: The 5-year survival rate based on acute exacerbation

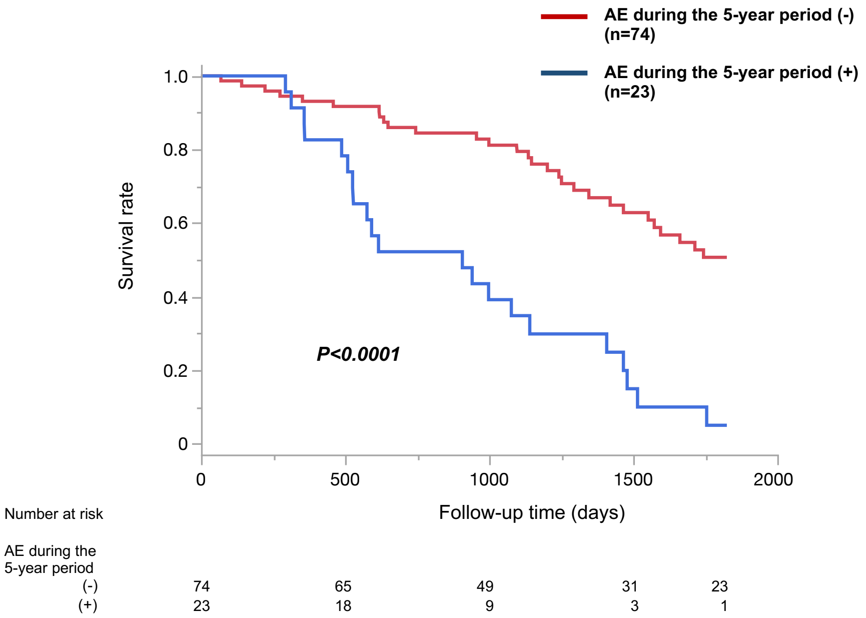

* *P*< 0.05, Spearman correlation coefficients

AE, acute exacerbation

S4: Association between baseline characteristics and serum sTLR4 levels

	sTLR4	
	r_s	*P*-value
Age, years	0.160	0.12
BMI, kg/m^2	-0.083	0.50
Smoking history, pack years	0.072	0.49
FVC, %	-0.073	0.48
WBC, /µL	0.130	0.21
Neutrophil, /µL	0.081	0.43
Lymphocyte, /µL	0.058	0.58
Monocyte, /µL	0.23	0.020*
Platelet, ×10^4 /µL	0.044	0.67
CRP, mg/dL	0.17	0.11

* $P< 0.05$, Spearman correlation coefficients

BMI, body mass index; CRP, C-reactive protein; FVC, forced vital capacity; sTLR4, soluble toll-like receptor 4; WBC, white blood cells

S5: Clinical characteristics of the study participants

	All patients with IPF (n=97)	Combination of sTLR4 and monocyte		
		Group A (n=17)	Group B (n=36)	Group C (n=44)
Age, years	68.0 (62.0−74.0)	68.0 (61.5−74.5)	69.0 (62.3−73.8)	67.0 (59.8−74.0)
Sex, male/female	86/11	17/0	33/3	36/8
BMI, kg/m^2	23.7 (21.6−26.2)	23.7 (22.8−25.2)	23.8 (21.4−25.6)	23.8 (21.5−27.0)
Smoking history, pack years	32.8 (15.9−49.5)	40.0 (22.0−60.0)	30.0 (18.8−40.0)	33.3 (11.3−49.5)
FVC, %predicted	74.3 (64.1−88.1)	76.4 (65.6−89.8)	74.3 (61.8−86.8)	73.6 (63.7−86.7)

Group A: Patients with soluble toll-like receptor 4 (sTLR4) level higher than 2.2 ng/mL and monocyte level higher than 381.0/μL

Group B: Patients with higher sTLR4 or higher monocyte levels

Group C: Patients with lower sTLR4 and lower monocyte levels

Data are presented as the median (interquartile range).

BMI, body mass index; FVC, forced vital capacity; IPF, idiopathic pulmonary fibrosis; sTLR4, soluble toll-like receptor 4
